# Measuring outcomes of community aged care programs: challenges, opportunities and the Australian Community Outcomes Measurement ACCOM tool

**DOI:** 10.1186/s12955-018-0918-7

**Published:** 2018-05-29

**Authors:** Beatriz Cardona

**Affiliations:** Macquarie University, Sydney, Australia

**Keywords:** Outcome measurement, Quality of life, ASCOT, ACCOM, Aged care

## Abstract

Measuring health and wellbeing outcomes of community aged care programs is a complex task given the diverse settings in which care takes place and the intersection of numerous factors affecting an individual’s quality of life outcomes. Knowledge of a strong causal relationship between services provided and the final outcome enables confidence in assuming the care provided was largely responsible for the outcome achieved (Courtney et al., Aust J Adv Nurs 26:49–57, 2009). The Department of Health has recently reported on the findings of The National Aged Care Quality Indicator Program – Home Care Pilot (KPMG, National Aged Care Quality Indicator Program – Home Care Pilot, 2017). The Program sought to test various tools to measure quality of life outcomes of their community aged care programs. Some of the key issues raised in the study reiterate the findings from The Australian Community Care Outcome Measurement (ACCOM) pilot study (Cardona et al., Australas J Ageing 36: 69–71, 2017), including the value of the ASCOT SCT4 tool (Adult Social care Outcomes Toolkit, http://www.pssru.ac.uk/ascot/downloads/questionnaires/sct4.pdf) to measure social care related quality of life (SCRQoL) in community aged care programs in the Australian context, the collection of additional data to map the relationship of various variables such as functional ability, demographic characteristics and quality of life scores and the governance and administration of measurement tools for the purpose of quality reporting and consumer choice.

## The aged care reforms and the role of outcomes in evaluation

The profound changes that have taken place in the aged care system in Australia have been rationalised as necessary to deliver better outcomes for older people and address the shortcoming of previous funding and service delivery models. The direction taken by the reforms was broadly set in the 2011 Productivity Commission Report, *Caring for Older Australians* [1–5]. The Australian Government responded to this report with the ‘Living Longer Living Better’ [6] reform package, which saw among other things, the introduction of a centralised entry point to aged care services, individualised funding models, and the transition from the Home and Community Care (HACC) and the Community Aged Care Packages into the Commonwealth Home Support Program (CHSP) and the Home Care Packages (HCP).

Individualised funding under Consumer Directed Care (CDC) model has replaced block funding for the HCP program in order to address a perceived lack of choice and flexibility on the type of programs and services available for older people. CDC has been described as increasing consumer choice and flexibility, and in turn, improving wellbeing including higher life satisfaction, greater life expectancy, independence and better continuity of care [5]. The move to marketisation and individualisation in aged care follows similar reforms in other social policy areas in Australia informed by public choice theories. Public choice theory proposes that markets should lead to higher efficiency, better quality, greater diversity and less bureaucracy in the delivery of public services [7].

Assessing the effectiveness of these reforms ultimately rests with the impact they have had on the individuals accessing the services. But how to measure this impact remains a challenge for various reasons. Impact indicators are multifaceted. Donabedian refers to three approaches to quality measurement and monitoring: structure, process and outcome [8]. In community aged care the structural measures assess inputs such as the number of staff available and training or the effectiveness of My Aged Care in providing accurate and timely information and pathways to service entry, etc., while process measures examine actual services or activities provided to consumers. Outcome measures are focused on identifying the impacts of these services on the health, wellbeing and quality of life of consumers.

Recent evaluations of My Aged Care for instance have identified increased consumer satisfaction with the information, interactions with the Contact Centre and linkages to services delivered through the online referral system [9]. A review of My Aged Care by the Legislated Review of Aged care in 2017 also identified various shortcoming with having a single entry point into aged care services which relies on consumers’ knowledge and skills in navigating the online website to search for services and choose providers. These formative evaluations, although important give us only a partial picture of the effectiveness of the reforms with the potential of prompting changes and improvements in processes to address its shortcoming. They however do not throw light on the key question of the effectiveness of the reforms in improving service outcomes for consumers.

## The feasibility and validity of various tools to measure outcomes and the ACCOM tool

To be able to demonstrate high standards and impact of services requires a focus on the outcomes of care – the results or consequences of the interventions assistance provided to older people through the aged care programs. This type of evaluation is however a complex undertaking, further complicated by uncertainty on the current draft clinical and wellbeing indicators that would guide the Single Aged Care Quality Framework to be implemented in July 2018 [10]. Furthermore, there is no current consensus as to what tools and methods should be used to measure the outcomes of services on consumers. A recent pilot study conducted by KPMG on behalf of The Department of Health sought to answer these questions by testing 4 outcome tools for community care. The National Aged Care Quality Indicator Program – Home Care Pilot [11] tested Goal Attainment Scaling (GAS) tool, the Adult Social Care Outcomes Tool SCT4 (ASCOT SCT4), which measures consumer experience and quality of life, Your Experience of Services (YES) Survey, which measures consumer experience and a combined tool based on two World Health Organisation Quality of Life questionnaires (WHOQOL-BREF (OLD)) which measures quality of life.

The study identified the ASCOT SCT4 tool as the most suitable instrument given its validity and useability. It also recommended further research on its applicability across multiple programs and the collection of demographic data to address impact of independent variables on outcomes. The collection of information on proxy responses and functional ability including cognitive capacity of respondents was also identified as important given their potential impact on response bias and response shift. The study also raised questions regarding the implementation and governance of the tool as well as future applicability in benchmarking given the voluntary character of service’ participation in outcome measurement recommended by the Department of Health.

The findings of the KPMG Care Pilot study in relation to the suitability of the ASCOT SCT4 tool and the need for the collection of additional data including demographic information support the findings of the Australian Community Care Outcome Measurement (ACCOM) pilot study [12]. The study, conducted through a collaborative and partnership research approach between the Australian Health Services Research Institute (AHSRI) at the University of Wollongong, Macquarie University, home and community aged care service providers and the University of Kent,   tested the use of a modified ASCOT SCT4 tool completed by consumers as well as case managers to measure social care related quality of life (SCRQoL) of consumers from multiple vantage points. The tool incorporated three key component measures identified for all consumers:

i. quality of life measures;
ii. functional measures of the capabilities and care needs; and
iii. data on the basic demographics and living conditions.

The use of the ASCOT was seen to have further benefits, including the fact that it has been extensively tested and revised, the validity and reliability of its scales is well known and the psychometric properties of its instruments found to be reliable [13]. The functional aspects are based on the HACC Functional Screen and include mobility, housework, shopping, medication management, financial management, personal care, and, if applicable, cognition and behavioural problems. It is a standardised instrument already used by most community care services and available data can be used to track the capability of consumers to perform activities of daily living (ADLs). It is key indicator of a care recipient’s needs for care and capacity to stay at home. Rather than requiring new data to be collected, the Functional Screen of the ACCOM is a standardised instrument that tracks the ability of consumers to perform activities of daily living (ADLs) over time. Functional ability (or capability) is a key indicator of a care recipient’s ability to stay at home [14]. For many progressive and chronic diseases, it is particularly important to understand functional abilities and limitations. Such information can assist service providers in understanding the needs of consumers around activities of daily living, their level of independence and the potential impact of these factors in their overall sense of wellbeing, control and quality of life.

The combination of the functional screen with the capabilities based framework of the ASCOT SCT4 four-level self-report version allowed a more comprehensive collection of health and social related QoL indicators reflecting the wider range of services provided in the community age care system in Australia. The Functional Screen tool also allowed testing the level of functioning relationship to the capabilities framework of the ASCOT SCT4 instrument, where the domains are phrased in the language of capabilities at the high quality of life end of the spectrum and in terms of functionings when reflecting lower quality of life.

Demographic data, including age, income, living circumstances and cultural background was also utilised in the ACCOM. This is already collected as part of service records for each consumer and updated where changes, such as death of spouse, occur. These additional elements enabled quality of life outcomes to be linked to the capabilities, care needs and demographic characteristics of consumers. The use of consumer’s and a case managers’ (CM) version of SCT4 is an innovative component of the ACCOM tool. It was introduced at the planning stage in response to feedback from case managers and community representatives who identified the importance of capturing not only consumers’ self- rating on QoL domains but also case managers’ perspectives on consumer outcomes. Research on self-reported outcomes has highlighted the value of multiple vantage points which can provide a more reliable estimate of change. Research on response shift in QoL appraisals supports the value of comparing self-reported change in QoL to other external measures of QoL change that are independent of consumer self-report including clinician judgment, performance tests, or family caregiver ratings.

**Fig. 1:**
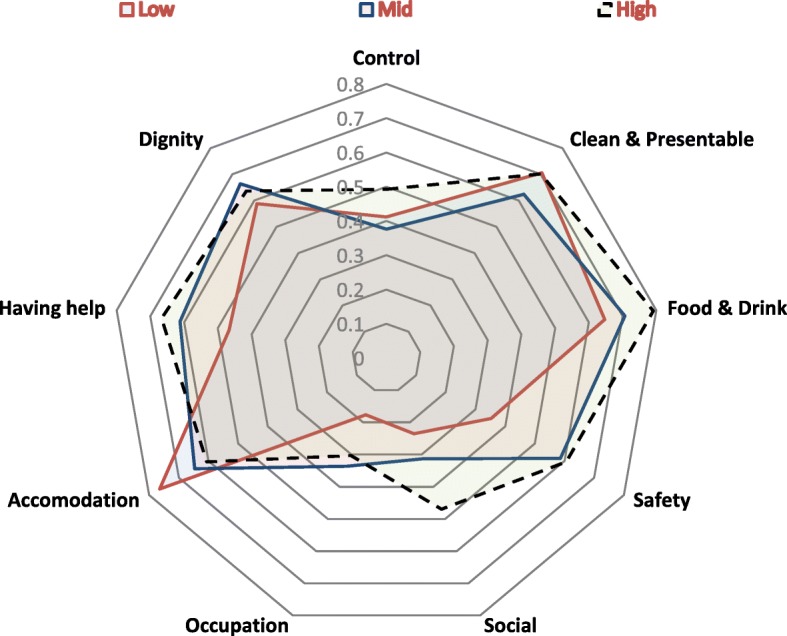
Consumers who selected the most positive response for each QoL Item by functional score in round 1 (*n* = 227)

Collecting additional information such as demographic data, was, as pointed out earlier, identified by the KPMG Home Care study, as important in order to ascertain attribution and account for factors that could potentially impact on quality of life scores. The use of the Functional Screen in the ACCOM study allowed the examination of links between the consumer responses to the SCRQoL domains and his/her functional capacity. For instance, as Fig. 1 indicates, in round 1 a higher proportion of those with high levels of functioning reported the most positive response possible in almost all the domains. While not unexpected, this suggests that QoL responses also reflect the personal capacity of each consumer to undertake tasks for her or himself [15].

**Fig. 2:**
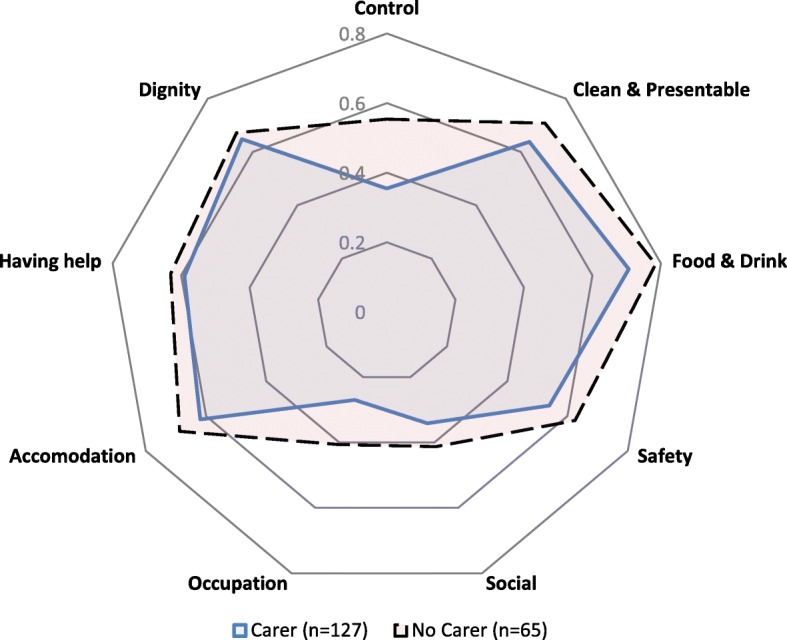
Consumers who selected the most positive response for each QoL Item by availability of carer in round 1

The collection of demographic data in the ACCOM tool allowed consideration of factors that could impact on QoL scores. For instance, the availability of an informal carer to provide support was shown to have affected QoL scores of consumers. In most cases the carer was a spouse, partner, daughter of other family member. As Fig. 2 indicates, there were slightly fewer consumers who selected the highest category of most QoL domains amongst those who had a carer than those without – a finding that at first seems unexpected. The explanation that seems most likely is that those who depend on carers are more likely to require high levels of ongoing assistance and be less capable of undertaking activities independently than those who are without a carer [12].

The introduction of a consumer as well as a case manager perspective on consumers’ QoL in the ACCOM tool was an innovative component of the study. It generated useful information on the value of multiple vantage points when measuring consumers SCRQoL. The ASCOT SCT4 tool was designed to collect the perspectives from consumers only, (it should be completed individually) and differs in this way from other forms of the ASCOT, such as those used in residential care, intended to be completed face to face by staff together with facility residents. It was interesting, for instance, to note the positive correlation between client and case manager Social Care Related Quality of Life Scores in round 1 (*r* = 0.408, *n* = 199). In round 2, the level of correlation was lower (*r* = 0.276, *n* = 118). The study identified the need for further analysis and testing of the ACCOM to investigate the extent to which differences in client and case manager responses reflect significant subjective differences or other considerations.

As the above discussion indicates, exploring the QoL data, derived from the ASCOT component of the ACCOM, and examining correlations with functional capabilities and demography can assist in understanding the links between the QoL experienced by consumers and factors that may influence it, such as the type and amount of services provided, the health and overall level of needs of the consumers concerned, as well as the availability and importance of informal social support.

Regarding the data collection and data submission, there are a few considerations worth noting. The KPMG Home Care Pilot recommended various strategies including the use of online data collection tools. This method, although effective in reducing administrative burden, could potentially limit the number of responses given the challenges some consumers face when using online tools. The ACCOM study recommended the use of paper based surveys with a self-return envelope sent to a centralised outcome evaluation centre which will enter, analyse and report on the data. Such process ensures the integrity and robustness of the system to enable service providers to demonstrate their ability to deliver high quality care that meets the health and quality of life needs of their consumers.

## Conclusion

Meeting the objectives of The National Aged Care Quality Indicator Program which are to give consumers transparent, comparable information about quality in aged care and for providers to have robust, valid data to measure and monitor their performance and support continuous quality improvement [16] is a complex and challenging task. The KPMG Home Care Pilot and the ACCOM study highlighted important methodological and governance considerations including the choice of instruments and the value of collecting functional and demographic data to explain possible links and factors accounting for quality of life scores and therefore be able to ascertain change or impact that can be attributed to service provision. The studies also called for caution before implementing nation-wide outcome measurement programs. The research is not yet conclusive regarding the appropriateness of the ACCOM and ASCOT tools for different cohorts including older people with cognitive impairment, from Culturally and Linguistically Diverse (CALD) backgrounds and Indigenous people.

There is also a need for further research looking at the appropriateness of the ASCOT tool for the Australian aged care context. Also we need further exploration of older Australian’s perceptions of what constitutes quality of life and the extent to which aged care services can support the achievement of these objectives. Research in Australia suggests that older adults value both health and social domains as important to their overall quality of life [17]. Further information is needed to ascertain the relationship between health status and wellbeing, especially when considering the compensatory rather than restorative character of services provided for older people at home.

Equally important there are still unresolved issues associated with the collection and management of the data. As mentioned earlier, it is essential to ensure the integrity and validity of the findings through the establishment of robust and independent systems to collect, analyse and report on the data. Such system would require investment in human capital and technological structures capable of exporting functional and demographic data collected during assessment and care planning.

More importantly, any outcome evaluation tools and findings need to be meaningful, and valid appraisals of the impact services have on the health, wellbeing and quality of life of consumers. They also need to provide services with useful and reliable information on the value and impact of their programs. This is particularly important when wellness, reablement and consumer choice is being legislated in program planning and delivery.

## Abbreviations

ACCOM: Australian community care outcomes measurement
ADL: Activities of daily living
ASCOT: Adult Social Care Outcomes Toolkit
CDC: Consumer directed choice
CHSP: Commonwealth Home Support Program
EQ-5D: EuroQol - 5 Dimensions
GAS: Goal attainment scale
HACC: Home and community care
HCP: Home Care Packages
ICECAP-O: ICEpop CAPability measure for Older people
SQRQoL: Social care related quality of life
WHOQoL-Old: World Health Organisation Quality of Life Instrument-Older Adults Module
YES: Your Experience of Services
